# Where Does Human Plague Still Persist in Latin America?

**DOI:** 10.1371/journal.pntd.0002680

**Published:** 2014-02-06

**Authors:** Maria Cristina Schneider, Patricia Najera, Sylvain Aldighieri, Deise I. Galan, Eric Bertherat, Alfonso Ruiz, Elsy Dumit, Jean Marc Gabastou, Marcos A. Espinal

**Affiliations:** 1 Department of Communicable Diseases and Health Analysis, Pan American Health Organization, Washington, D.C., United States of America; 2 Unit of Control of Epidemic Diseases, World Health Organization, Geneva, Switzerland; 3 Department of Global Health, University of South Florida, Tampa, Florida, United States of America; University of California San Diego School of Medicine, United States of America

## Abstract

**Background:**

Plague is an epidemic-prone disease with a potential impact on public health, international trade, and tourism. It may emerge and re-emerge after decades of epidemiological silence. Today, in Latin America, human cases and foci are present in Bolivia, Brazil, Ecuador, and Peru.

**Aims:**

The objective of this study is to identify where cases of human plague still persist in Latin America and map areas that may be at risk for emergence or re-emergence. This analysis will provide evidence-based information for countries to prioritize areas for intervention.

**Methods:**

Evidence of the presence of plague was demonstrated using existing official information from WHO, PAHO, and Ministries of Health. A geo-referenced database was created to map the historical presence of plague by country between the first registered case in 1899 and 2012. Areas where plague still persists were mapped at the second level of the political/administrative divisions (counties). Selected demographic, socioeconomic, and environmental variables were described.

**Results:**

Plague was found to be present for one or more years in 14 out of 25 countries in Latin America (1899–2012). Foci persisted in six countries, two of which have no report of current cases. There is evidence that human cases of plague still persist in 18 counties. Demographic and poverty patterns were observed in 11/18 counties. Four types of biomes are most commonly found. 12/18 have an average altitude higher than 1,300 meters above sea level.

**Discussion:**

Even though human plague cases are very localized, the risk is present, and unexpected outbreaks could occur. Countries need to make the final push to eliminate plague as a public health problem for the Americas. A further disaggregated risk evaluation is recommended, including identification of foci and possible interactions among areas where plague could emerge or re-emerge. A closer geographical approach and environmental characterization are suggested.

## Author Summary

Plague is a disease of epidemic potential that could emerge and re-emerge after decades of epidemiological silence. Today, in Latin America, human cases and natural foci are present in Bolivia, Brazil, Ecuador, and Peru. We searched for official information of where cases of human plague still persist in Latin America, mapped the areas, and briefly described selected factors. This analysis will provide evidence-based information for countries to prioritize areas for intervention. A geo-referenced database and risk map were created. One or more events of plague were found in 14 of 25 countries in Latin America in the period of 1899–2012. There is evidence that human cases of plague still persist in 18 of the almost 13,300 second levels of the political/administrative divisions (counties). Demographic and poverty patterns were observed in 11/18 counties. Four types of biomes are most commonly found and 12/18 counties have an average altitude higher than 1,300 meters above sea level. Even though human plague cases are very localized, the risk is still present, and unexpected outbreaks could occur. Countries need to make the final push to eliminate plague as a public health problem for the Americas. A further disaggregated risk evaluation and a closer environmental characterization are recommended.

## Introduction

Plague is a historic disease that is responsible for some of the most devastating epidemics in human history. Despite its low and decreasing incidence, it continues to be endemic in some countries of the world and remains a public health threat. Plague is an epidemic-prone disease with a high case-fatality rate, which can generate panic in the population, induce political tension, and impact public health systems, along with international trade and tourism. Recent plague outbreaks illustrate the capacity of this disease to re-emerge after a long quiescent period. Examples include cases in Oran, Algeria in 2003 after more than 50 years and in Mbulu, Tanzania in 2007 and 2008 after more than 30 years [1], [2].

Plague is a zoonotic disease caused by the bacterium *Yersinia pestis* that involves primarily wild rodents (reservoir) and their fleas (vectors), which occasionally transmit the disease to other animals and humans, particularly those living near natural disease foci [3]–[6]. It is an excellent example to illustrate the human-animal-ecosystem interactions and the One Health framework could be used to address health risks at this interface [7], [8]. Plague infection can manifest in humans in three clinical forms, depending on the route of exposure: bubonic, septicaemic, and pneumonic. As per the International Health Regulations (IHR) (2005), pneumonic plague is one of the notifiable diseases listed as a potential Public Health Emergency of International Concern (PHEIC) [9].

The first well-known worldwide pandemic happened in the Justinian period (around 541 A.D.) and spread around the Mediterranean Sea. The second worldwide pandemic was registered from the 14^th^ to the 16^th^ centuries, affected Central Asia, Africa and Europe, and was denominated “Black Death”, causing a great loss of life. Lastly, the third and more recent pandemic originated in China during the 19^th^ century [10], [11].

It was during the third and last pandemic that plague occurred for the first time in the Region of the Americas (along with West Africa, South Africa, Madagascar and Indochina), beginning in port cities where it was probably introduced by sea or riverboat traffic [3], [10]. After the introduction of plague to the harbor cities, large human outbreaks occurred in densely populated urban centers. The infection gradually propagated inland, generally following transportation routes. As domestic rats intermingled with wild animals in rural areas, the infection was transmitted to these wild hosts, which today serve as the main reservoir in areas where plague remains a public health problem [10]. In the Region of the Americas, several rodents have been identified as reservoirs, such as *Akodon* sp, *Oryzomys* sp, and *Rattus*. The main vector is the rodent flea *Xenopsylla cheopis* [4].

Plague is considered to be endemic in 26 countries around the world, but most cases are found in remote areas of Africa [12]. Between 2004 and 2009, 16 countries in Africa, Asia, and the Americas reported human cases of this disease. In this time period, there was an average of around 2,000 cases per year, with more than 90% of them reported in Madagascar and the Democratic Republic of Congo [12]. The incidence decreased substantially after 2009. According to the World Health Organization (WHO), 400 human cases of plague and 75 deaths were recorded in 2012, mainly in Africa [13].

In some geographic areas in the American Region considered as foci, plague appears to be maintained in nature by transmission among local susceptible rodents and flea fauna. In Latin America, human cases of plague and natural foci without evidence of human cases are present in Bolivia, Brazil, Ecuador and Peru [14]. In the western region of the United States, human cases of plague also continue to occur in rural and semi-rural areas where many types of rodent species can be involved in the plague foci [15]–[17]. In the Americas, countries that are considered endemic for plague have been conducting surveillance and implementing prevention and control measures [18]–[21].

Understanding the environmental influences in a particular geographical region where plague currently occurs is important for disease prediction, prevention and control measures [22]. Several studies have been conducted to evaluate the environmental influences in the occurrence of plague in order to better understand the complex cycle of this disease and to predict areas of potential transmission [23]–[27]. Some of the studies analyzed the relationship between precipitation and temperature with plague, while others looked at altitude, soil, and other conditions [28]–[30]. In Latin America, plague generally strikes impoverished communities in remote rural areas [31].

The main objective of this study is to identify where cases of human plague still persist in Latin America and map areas that may be at risk for disease emergence or re-emergence. Knowing when a case is unusual or unexpected is part of the verification and response of event management within the IHR. This information could also provide evidence-based information for decision makers to prioritize areas to prevent, detect, and respond to possible outbreaks of plague.

## Methods

### Study design

The first part of the study was a retrospective search for the historical presence of plague in Latin America. The information obtained was consolidated in order to determine where human cases of plague still persist and the potential areas where it could re-emerge. Scientific publications about plague re-emergence were used to define the time periods for the analysis.

Based on this information, it was possible to describe the historic presence of plague in Latin America, by country and year, from the appearance of the first documented case in 1889 until 2012. The different steps used in the study's data collection and analysis are described in Table 1.

**Table 1:** Study steps.

Steps	Actions taken
1	Recover evidence of the presence of human plague by reviewing existing official information from WHO, the Pan American Health Organization (PAHO), and Ministries of Health of Latin America. No time period was established.
2	Create a table with the information obtained by year and by country, marking the year where evidence was found and the source (1899–2012). When more than two sources were found for that specific information, only the first two references were included in the table. Any countries in Latin America (total of 25 countries) that appeared at least once in all the sources reviewed were included in the table (Supporting Information S1 and S2).
3	Review the data that was found and decide what level of desegregation by period could be done with the information available. Review scientific publications about plague re-emergence to define the time periods for the analysis (1899–1949; 1950–1979; 1980–2012). Create definitions and time space units of analysis to be used in this study and next steps for the analysis.
4	Create a geo-referenced database with the table information by country. Perform historical and geographical analysis of human plague persistence by country using the time periods defined. The total number of years with presence of disease by each time period was added up to measure the length of persistence.
5	Review the information and create tables and a map to identify where human plague still persists according to the definition created for this study, which is by county (2000–2012). The sources are described in detail in the Supporting Information S3. Develop a graph with the number of human cases of plague by year and by country from 2000–2012.
6	Create a second geo-referenced and geo-coded database with information where plague still persists.- Process environmental data to calculate the extent and delineate biomes and soils within productive areas using a geographic overlapping technique -named geo-processing/intersect- overlay- Perform spatial analysis -zonal statistics technique- to measure, assign and quantify productive areas' altitude, slope, temperature and precipitation. Include this as numeric information in the database and tables.
7	Collect selected demographic and socioeconomic variables from the productive areas (sources are described in detail in the Supporting Information S4).
8	Describe the productive areas characteristics' with selected demographic, socioeconomic and environmental variables that may be related to presence of plague based in scientific literature review.
9	Create a table and a map that include the areas where plague still persists and where it could potentially emerge or re-emerge (Supporting Information S5). This was the most desegregated level that the information was available.
10	Add to the risk map layers with additional information about the location of major roads, rivers and railroads to analyze possible overlap with the productive areas.

Within the currently endemic countries, two parallel analyses were performed by identifying: 1) Areas “where plague still persists” at the second level of the political/administrative divisions that are called municipalities, cantons, or provinces in different countries, but for the purpose of this study are called counties; 2) Potential areas where it could emerge or re-emerge at the first level of the political/administrative divisions that are designated as departments, states, or provinces in different countries, but for the purpose of this study are called regions. This was the most disaggregated level for which information was obtained.

Maps were produced to demarcate spatially the counties where plague still persists in order to be used as a baseline for the current situation. Based on the literature review, selected demographic, socioeconomic, and environmental variables were used to describe the counties where plague still persists and explore ideas for future more disaggregated studies. Lastly, a geographic analysis was carried out to map the risk for plague in Latin America, including counties where plague still persists and regions where it could emerge or re-emerge. The location of major roads, rivers, ports, and airports were also included in the map to determine whether they overlap with the counties where plague still persists.

### Definitions

#### Human cases of plague

The presence of plague in Latin America was defined according to whether or not there was evidence of human cases in a certain time period or year, based on the fact that the number of cases was not always available and that the definition of plague cases could have changed in the time period and between countries. The case definition for confirmed plague cases according to the WHO guidelines is: “isolate identified as *Y. pestis* by phage analysis of cultures; or a significant (4-fold) change in antibody titre to the F1 antigen in paired serum specimens” [17]. It was expected that for the period between 2000 and 2012 the countries used this definition.

#### Periods for plague emergence or re-emergence

For the analysis at country level for the entire period which information was obtained (1899–2012), the following cuts were included in the timeline based on scientific publications [1], [2]:

- Countries considered out of the window of potential re-emergence: Presence of cases of human plague more than 60 years ago (from 1889 to 1949).
- Countries considered within the window of potential re-emergence: Presence of cases of human plague between 30 and 60 years ago (from 1950 to 1979).
- Countries considered as endemic: Presence of cases of human plague in the last 30 years (from 1980 to 2012)

#### Epidemiological characterization of the endemic countries

Counties where plague still persists:

- Productive areas where plague still persists: political/administrative divisions (in this case, at the second sub-national level) where evidence of the presence of cases of human plague was detected in official documents between 2000 and 2012.

Regions with potential risk of plague emergence and re-emergence:

- Endemic regions with productive areas: political/administrative divisions (in this case, at the first sub-national level) where evidence of the presence of cases of human plague was detected between 2000 and 2012.
- Endemic regions in epidemiological silence: political/administrative divisions (in this case, at the first sub-national level) that have reported one or more cases of human plague between 1980 and 1999 and where there is no evidence of the presence of cases of human plague between 2000 and 2012.
- Regions with foci: political/administrative divisions (in this case, at the first sub-national level) that have been cited in the literature between 1980 and 2012 with the presence of one or more foci and no evidence of human cases in the same period. No specific definition of foci was used, if it was reported as foci, it was included in our database.

### Data collection

#### Data on the historical presence of cases of human plague in Latin America by country

In order to obtain information about the evidence of human plague cases, only official databases and documents were consulted: WHO Weekly Epidemiological Bulletin, PAHO Epidemiological Bulletin, Health in the Americas, PAHO Institutional Memory Database that contains historical documents, WHO and PAHO online databases and documents from those webpages. In addition, official online databases, Epidemiological Bulletins, and documents from the webpages of the Ministries of Health of Bolivia, Brazil, Ecuador and Peru were searched. Presentations from the endemic countries' representatives during the “International Meeting of Plague Experts in Latin America”, organized by PAHO in 2013, were also used (PAHO, unpublished data).

Based on the information obtained, a database of cases of human plague by year and country was created using many different official sources [6], [20], [21], [32]–[47] (Pezantes C, unpublished data) (Supporting Information S1 and S2). Latin America has 25 countries and territories according to the official PAHO geographic configuration [48]. Countries that appeared at least once in all the sources reviewed were included in the geo-referenced database and references were cited in the table. When more than two sources were found for that specific information, only the first two references were included in the table (Supporting Information S1).

#### Data on the presence of human cases of plague and the presence of natural foci in Latin America by region

The time period for this analysis was 1980 to 2012. The same sources were reviewed and additional information was obtained about human cases by region. The existence of plague foci where no human cases were reported in that period were included in a corresponding GIS database disaggregated by region of the endemic countries.

#### Data on the evidence of where human plague still persists in Latin America by county - productive areas

The time period for this analysis was 2000 to 2012. The main sources were: (1) Epidemiological Bulletins and online databases from the Ministries of Health of endemic countries, when information was available; (2) presentations from endemic countries' representatives during the “International Meeting of Plague Experts in Latin America” organized by PAHO; and (3) previous information sent by country officials directly to PAHO when plague was a disease of mandatory reporting until June 2007, when the new version of the IHR became official. Specific sources by country are described in the Supporting Information S3.

#### Demographic and socioeconomic data for the productive areas

The total population and the percentage of rural population were obtained mostly from the countries' census [49]–[53]. Poverty-related variables were obtained for each country; however, indicators vary among countries [54]–[60]. For this reason, poverty can only be compared within countries. The national and regional values were included to facilitate the comparison with the counties. Specific sources by country for each demographic and socioeconomic variable are available in the Supporting Information S4: Table S1.

#### Environmental data for the productive areas

For the productive areas, a number of environmental variables were processed to characterize and identify the predominant geographical features. Altitude (meters above sea level) and (degrees 0–90) were obtained from the U.S. Geological Survey's (U.S.G.S.) EROS Data Center developed in collaboration with the United Nations Environment Program/Global Resource Information Database (UNEP/GRID) HYDRO1k and processed from the original grid format [61].

Biomes or terrestrial eco-regions cartography was obtained from ESRI/World Wildlife Fund [62]. Biomes are defined as relatively large areas of land or water containing a characteristic set of natural communities that share a large majority of their species, dynamics, and environmental conditions. Bioclimatic variables –Bioclim- were obtained from the Worldclim database [63], [64]. Specific sources for each environmental variable are available in the Table S1 in Supporting Information S4. For the purpose of this study, we selected:

- BIO1: Annual Mean Temperature (the mean of all weekly mean temperatures). Each weekly mean temperature is the mean of that week's maximum and minimum temperature in degree Celsius.
- BIO12: Annual Precipitation (the sum of all monthly precipitation estimates in millimeters)

The digital soil map of the world was used to identify soil types and groups and to measure their overlap with the productive area [65].

### Cartography and geo-processing analysis

The digital cartographic data for Latin America were collected, updated, standardized and geo-processed to connect and overlap with the environmental data, using ArcGIS/Editor 10.1 by PAHO CHA/IR/GIS working group. The first step was to update the political/administrative boundaries available from the PAHO-UN SALB Project, following the boundary depiction compiled since 2007 in the context of the activities of the UN Geographic Information Working Group (UNGIWG) [66].

Diverse techniques using Geographic Information Systems (GIS) were applied to select units of analysis in order to classify them and to link the areas with their environmental factors:

- A digital database of the Latin American countries was prepared to identify the presence of plague and categorize the number of years of plague presence by country.
- A digital database by county was created to characterize the presence of plague and the ecological conditions.
- Geographic proximity techniques were used to identify the productive areas' contiguous neighbors.
- Environmental digital cartography was processed and prepared to overlap with the county digital database:
  - An overlapping technique (named geo-processing/intersect) was used to delineate and calculate the extent of biomes and soils within the productive areas. This information was included in the numeric database.
  - Spatial analysis (zonal statistics technique) was used to measure, ascribe, and quantify the altitude, slope, temperature, and precipitation of plague productive areas. These data were included in the numeric database.
- A digital database by region was prepared to include the endemic attributes and overlay with the productive areas.

## Results

### Presence of plague

#### Historical revision by country - From 1899 to 2012 (entire time period)

According to the references reviewed, plague was first introduced in Latin America in Paraguay in 1899, followed by Brazil and Argentina. The presence of plague during one or more years was found in 14 out of 25 countries and territories in Latin America. Table 2 shows the 14 countries with presence of cases of human plague in Latin America by quinquennium. During this time period, the presence of plague in one specific year was found in three countries (Paraguay, Panama, and El Salvador); during two years in three countries (Cuba, Mexico and Puerto Rico); and during three years in two countries in this time period (Chile and Uruguay) (Table 3).

**Table 2:** Countries with presence of cases of human plague, by quinquennium, Latin America, 1899–1949.

Year	Countries													
	Argentina	Bolivia	Brazil	Chile	Cuba	Ecuador	El Salvador	Mexico	Panama	Paraguay	Peru	Puerto Rico	Uruguay	Venezuela
1899–1904*	X		X	X				X		X	X		X	
1905–1909	○		X			X			X		X			X
1910–1914	X		○		X	X					X	X		X
1915–1919	○		○		X	X					X			X
1920–1924	○	X	○			X		X			X	X		○
1925–1929	○	X	X			X					X		X	X
1930–1934	X	X	X	X		X					○		X	X
1935–1939	X	X	X			X					X			X
1940–1944	X	X	X			X					○			X
1945–1949	X	X	X			X					X			X
1950–1954	X	X	X			X					X			X
1955–1959	X	X	X			X	X				X			X
1960–1964		X	X			X					X			X
1965–1969		X	X			X					X			
1970–1974		X	X			X					X			
1975–1979		X	X			X					X			
1980–1984		X	X			X					X			
1985–1989		X	X			X					X			
1990–1994		X	X								X			
1995–1999		X	X			X					X			
2000–2004		X	X			X					X			
2005–2012*		X!	X			X					X			

**Legend:**

X: Presence of plague documented on that quinquennium; ○: Presence of plague documented on the period (consolidated data);

! : Suspected case;

* First and last quinquennium years added.

**Table 3:** Timeframe and presence of plague history, Latin America.

Period	Presence of plague in countries
1899–1949	First case reported in Latin America: 1899 (Paraguay*).First cases reported in countries: Brazil (1899); Argentina (1899); Uruguay (1901); Mexico (1902); Chile(1903); Peru (1903); Panama (1905*); Ecuador (1908); Venezuela (1908); Cuba (1912); Puerto Rico (1912); Bolivia (1921).Last cases reported in countries: Cuba (1915); Puerto Rico (1921); Mexico (1923); Chile (1931); Uruguay (1932).Summary of the situation: In two countries plague was present only in one year; in three countries twice; in two countries in three years; in six countries plague persists in foci and presented human cases in many years consecutive.
1950–1979	Case reported in El Salvador (1955*).Last cases reported in countries: Argentina (1958); Venezuela (1963).Summary of the situation: Two countries do not have more evidence of human cases, in the other four countries plague persists. Argentina and Venezuela are close to the timeframe of potential re-emergence of the disease.
1980–2012	Last cases reported in countries: Bolivia (2012!); Brazil (2005); Ecuador (2008); Peru (2012).Summary of the situation: Four countries considered as endemic, and in all human plague still persist (from 2000 to 2012).

**Legend:**

* : First and last case reported;

! : Suspected case;

The disease persisted for more than fifty years in six countries: in Argentina, the first case was in 1899 and the last case in 1958; in Venezuela, the first case was in 1908 and the last case in 1963; in Bolivia, the first case was in 1921 and the last case in 2012 (still persists); in Brazil, the first case was in 1899 and the last case in 2005 (still persists); in Ecuador, the first case was in 1908 and last case in 2008 (still persists); in Peru, the first case was in 1903 and the last case in 2012 (still persists).

**Figure 1:**
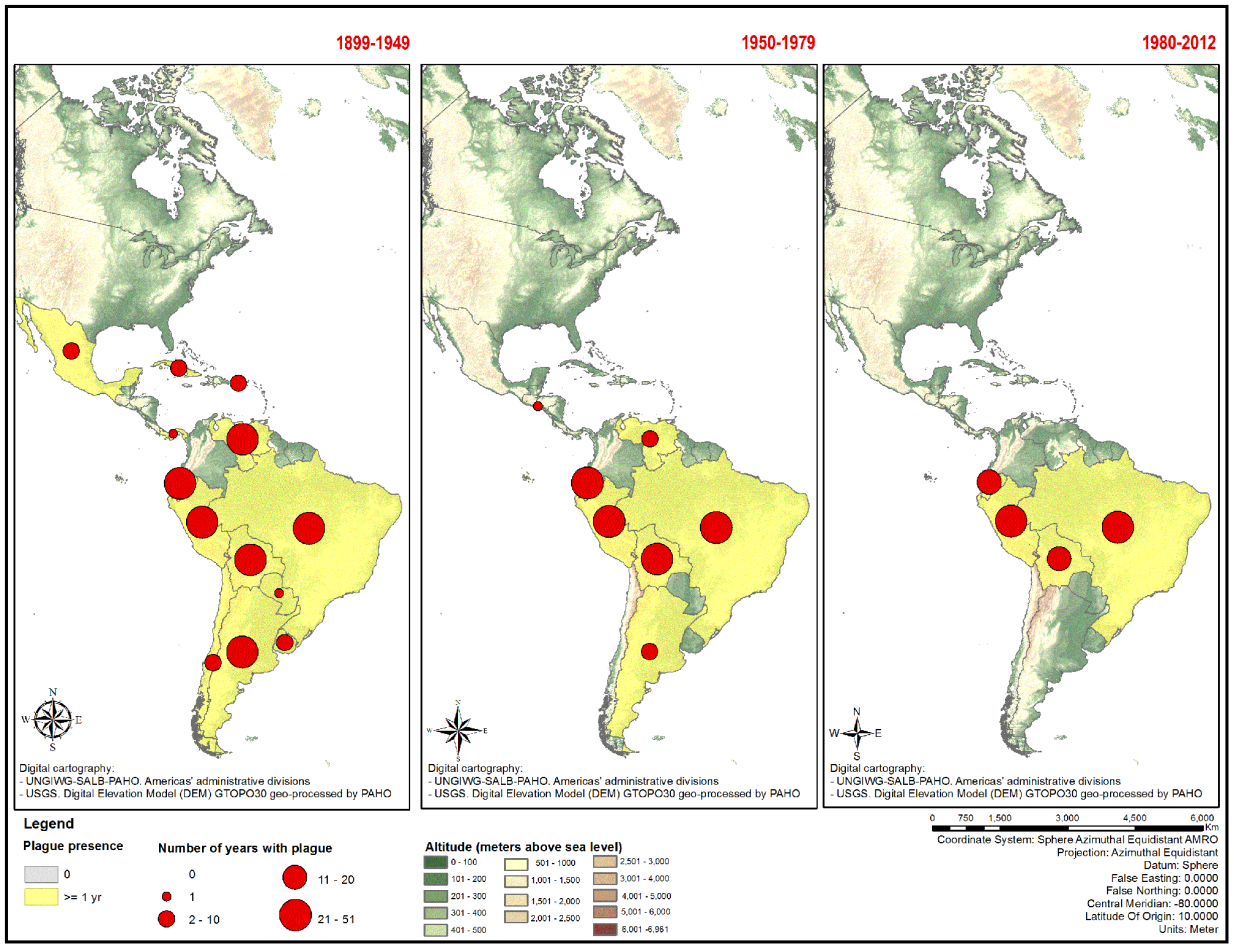
Historic information of presence of human cases of plague in Latin America, 1899–2012.

In Figure 1, a series of maps shows the historical information on the presence of human cases of plague in Latin America for the entire period, represented by the total number of years plague was present in each time period. The breaks in time based on the literature review show:

- Countries considered to be out of the window of potential re-emergence (presence of human plague cases more than 60 years ago, 1899–1949) were Cuba, Chile, Mexico, Panama, Paraguay, Puerto Rico, and Uruguay – all with isolated events. According to the analysis presented in Figure 1, the six countries (Argentina, Bolivia, Brazil, Ecuador, Peru and Venezuela) that presented 21 or more years of presence of plague during this period (1899–1949) continue to present human cases in the following years, suggesting that the disease was maintained in nature as foci.
- Countries considered to be in the window of potential re-emergence (presence of human plague cases from 30 to 60 years ago, 1950–1979) were Argentina and Venezuela. These countries had evidence of prolonged persistence of the disease in humans through much of the first five decades. The four countries (Bolivia, Brazil, Ecuador, and Peru) that presented 21 or more years of presence of plague during the period (1950–1979) continue presenting human cases in the following years. There was only one isolated event in El Salvador in this period, and for this reason, it was not considered in the window of potential plague re-emergence.
- Countries considered as endemic (presence of human plague cases in the last 30 years, 1980–2012) were Bolivia, Brazil, Ecuador and Peru.

#### Endemic countries - From 1980 to 2012 by region (last 30 years)

Fifteen out of around 580 regions (first level of the political/administrative divisions) in Latin America, or about 2.3%, show documented presence of human plague cases. Evidence of plague was found in two out of nine regions in Bolivia (La Paz and Santa Cruz), six out of 27 in Brazil (Bahia, Ceara, Minas Gerais, Paraiba, Pernambuco, Rio Grande do Norte), three out of 22 in Ecuador (Chimborazo, Cotopaxi, Loja), and four out of 25 in Peru (Cajamarca, La Libertad, Lambayeque, and Piura) (Supporting Information S5). Foci were cited in the literature in regions that did not report human cases in Bolivia (Chuquisaca and Tarija) and in Brazil (Alagoas, Piaui, Rio de Janeiro). In Ecuador and Peru, all regions with information reported human cases in this period.

#### Areas where human plague still persists - From 2000 to 2012 by county (last decade)

**Figure 2:**
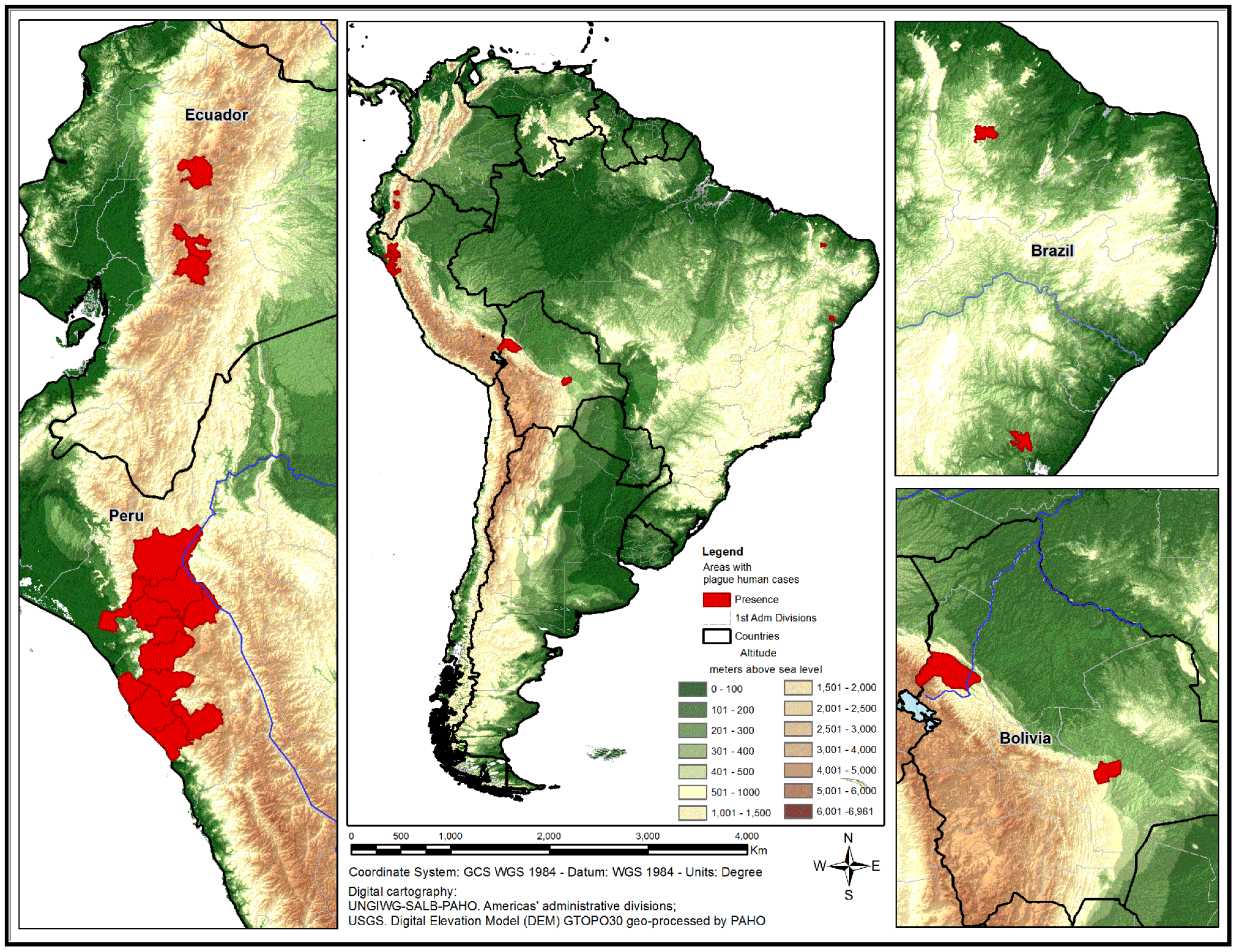
Counties with presence of cases of human plague, Latin America, 2000–2012.

It was observed that plague still persists in 18 counties (second level of the political/administrative divisions): two in Bolivia (Franz Tamayo and Andres Ibãnez), two in Brazil (Feira de Santana and Pedra Branca); three in Ecuador (Guamote, Riobamba, Latacunga), and in 11 contiguous counties in Peru (Chota, Contumaza, Cutervo, Jaen, San Miguel, Santa Cruz, Ascope, Otuzco, Pacasmayo, Trujillo, Ferrenafa). Evidence of plague in 18 out of about 13,300 counties in Latin America suggests that the disease is very localized in the Region (Figure 2). These 18 productive areas share borders with 70 other counties.

**Figure 3:**
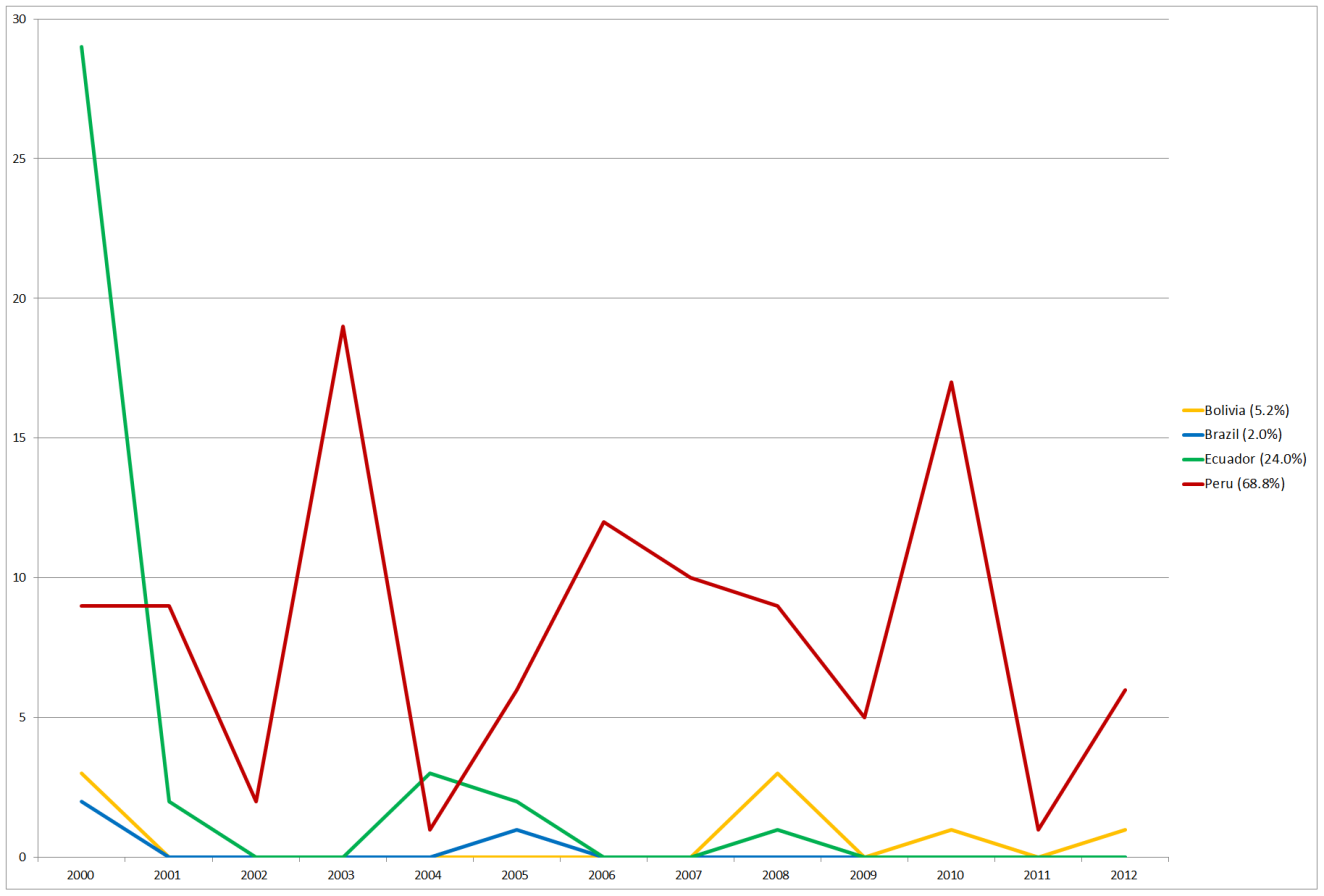
Number of human cases of plague, by country, Latin America, 2000–2012.

There were a total of 154 cases in Latin America in this period (eight in Bolivia, three in Brazil, 37 in Ecuador, and 106 in Peru) (Figure 3). Of the total number of cases, 68.8% were reported in Peru. Most of them were reported in Cajamarca before 2009, and all of them were reported in La Libertad between 2009 and 2012. The second largest percentage of cases was in Ecuador (24.0%), with 29 out of 37 cases in 2000 in Chimborazo.

#### Selected demographic, socioeconomic and environmental variables in counties where plague still persists -productive areas

A selection of variables was used to describe the areas where plague still persists. Demographic and poverty patterns were observed in 11 of the 18 productive areas, presenting a population of less than 200 thousand inhabitants, 40% of which are rural with high poverty rates (Table 4). However, some productive areas do not follow this pattern and have large, mostly urban populations with low rates of poverty.

**Table 4:** Demographic and socioeconomic characteristic of sub-national divisions with presence of plague, Latin America, 2000–2012.

Country	Regions	Counties	Total population	Rural population (%)	Poverty related (%)
Bolivia			10,426,155	37.6	40.4
	La Paz		2,839,946	33.9	42.4
		Franz Tamayo	19,347	88.5	75.6
	Santa Cruz		2,785,762	23.8	25.1
		Andres Ibanez	1,841,282	6.5	12.1
Brazil			190,755,799	15.6	33.7
	Bahia		14,016,906	27.9	43.5
		Feira de Santana	556,642	8.3	36.1
	Ceara		8,452,381	24.9	53.9
		Pedra Branca	41,890	41.5	68.6
Ecuador			14,483,499	37.2	60.1
	Chimborazo		458,581	59.2	66.5
		Guamote	45,153	94.1	95.5
		Riobamba	225,741	35.2	46.5
	Cotopaxi		409,205	70.4	75.1
		Latacunga	170,489	62.6	64.7
Peru			27,412,157	24.1	11.5
	Cajamarca		1,387,809	67.3	24.9
		Chota	160,447	79.9	28.6
		Contumaza	31,369	57.6	29.8
		Cutervo	138,213	80.6	25.8
		Jaen	183,634	49.9	21.9
		San Miguel	56,146	83.8	22.0
		Santa Cruz	43,856	79.0	26.6
	La Libertad		1,617,050	24.6	12.9
		Ascope	116,229	12.0	7.7
		Otuzco	88,817	76.2	29.5
		Pacasmayo	94,377	7.1	6.4
		Trujillo	811,979	2.4	3.0
	Lambayeque		1,112,868	20.5	6.5
		Ferrenafe	811,979	2.4	15.8

Twelve out of the 18 productive areas have an average altitude higher than 1,300 meters above sea level (Table 5). The Andean countries show contrasting altitudes compared to Brazil. The Bolivian region of Franz Tamayo registers a maximum altitude of 5,811 meters, while Feira de Santana in Bahia, Brazil reaches only 552 meters. The highest average altitudes are found in Ecuador.

**Table 5:** Environmental characteristics of sub-national divisions with presence of plague, Latin America, 2000–2012.

Country	Regions	Counties	Altitude (m)			Slope (degrees)			Temperature (°C)			Precipitation (mm)		
			Min	Mean	Max	Min	Mean	Max	Min	Mean	Max	Min	Mean	Max
Bolivia	La Paz	Franz Tamayo	225	2798	5811	0	7	37	−3	13	25	628	1238	2295
	Santa Cruz	Andres Ibanez	284	562	1679	0	2	16	19	24	25	726	1136	1364
Brazil	Bahia	Feira de Santana	115	208	552	0	1	9	21	23	24	663	892	1209
	Ceara	Pedra Branca	240	521	805	0	2	9	22	24	27	625	762	886
Ecuador	Chimborazo	Guamote	2785	3589	4422	0	7	19	3	8	13	445	706	1097
		Riobamba	2391	3521	4997	0	8	27	0	9	16	550	803	1150
	Cotopaxi	Latacunga	2690	3563	5682	0	6	24	−4	8	14	506	845	1207
Peru	Cajamarca	Chota	232	2311	3931	0	10	33	7	15	24	119	807	1293
		Contumaza	162	1513	3975	0	10	28	7	17	22	16	354	909
		Cutervo	426	2194	3852	0	11	33	7	16	25	378	822	1124
		Jaen	356	1790	3918	0	11	28	7	19	25	375	779	1456
		San Miguel	224	2306	4080	0	7	31	6	14	23	48	634	1552
		Santa Cruz	379	2395	3838	0	9	30	8	14	23	144	835	1423
	La Libertad	Ascope	4	455	2390	0	4	22	14	20	21	5	78	488
		Otuzco	553	3131	4220	0	10	33	5	11	20	129	630	1139
		Pacasmayo	6	1205	2223	0	5	22	14	18	23	6	243	460
		Trujillo	0	724	3615	0	7	26	8	18	20	3	99	431
	Lambayeque	Ferrenafe	24	1556	3931	0	6	26	7	18	23	27	496	1175

Steep slopes (above 20 degrees of inclination) are common in Andean productive areas, except for Andres Ibanez in Santa Cruz, Bolivia. On the other hand, in the Brazilian productive areas, the maximum slope is below 11 degrees. The annual precipitation is as low as 3 mm in Ascope, La Libertad, Peru, in contrast with a maximum of 2,295 mm found in Franz Tamayo, La Paz, Bolivia. Considering groups of productive areas, La Libertad in Peru was the driest area (3 mm–431 mm in Trujillo) while Santa Cruz, Bolivia presented the most humid conditions (726 mm–1364 mm in Andres Ibanez). The lowest temperatures and widest range were found in Ecuador (altogether minimum, mean, and maximum temperatures); while the highest temperatures were found in the Brazilian productive areas that, in turn, have very little variation among them.

A total of five types of biomes are present in the productive areas. Biomes that could be considered dry include: montane grasslands and shrublands (MGS) present in 12 out 18 productive areas; tropical and subtropical dry broadleaf forests (TSDBF) in 10; deserts and xeric shrublands (DXS) in nine; and tropical and subtropical grasslands, savannas and shrublands (TSGSS) in one area. Tropical and subtropical moist broadleaf forests (TSMBF), which could be considered a humid biome, are present in 11 out of 18 productive areas (Table 6).

**Table 6:** Biomes and soil types of sub-national divisions with presence of plague, Latin America, 2000–2012.

Country	Regions	Counties	Biomes					Soil types							
Bolivia	La Paz	Franz Tamayo	TSDBF		MGS			LP	RG	CM					LV
	Santa Cruz	Andres Ibanez	TSDBF	TSGSS			TSMBF	LP		CM			FR		
Brazil	Bahia	Feira de Santana				DXS	TSMBF							PH	AC
	Ceara	Pedra Branca				DXS								PH	LV
Ecuador	Chimborazo	Guamote			MGS		TSMBF	LP				AN		PH	
		Riobamba			MGS		TSMBF	LP				AN		PH	
	Cotopaxi	Latacunga			MGS		TSMBF					AN		PH	
Peru	Cajamarca	Chota	TSDBF		MGS		TSMBF	LP	RG	CM					
		Contumaza	TSDBF		MGS	DXS	TSMBF	LP	RG	CM	AR				
		Cutervo	TSDBF		MGS			LP	RG	CM					
		Jaen	TSDBF		MGS		TSMBF	LP	RG	CM					
		San Miguel	TSDBF		MGS	DXS	TSMBF	LP	RG	CM					
		Santa Cruz	TSDBF		MGS		TSMBF	LP	RG	CM					
	La Libertad	Ascope				DXS		LP			AR				FL
		Otuzco			MGS	DXS	TSMBF	LP	RG	CM					
		Pacasmayo	TSDBF			DXS		LP			AR				FL
		Trujillo				DXS		LP			AR				FL
	Lambayeque	Ferrenafe	TSDBF		MGS	DXS		LP	RG		AR				

**Legend:**

**Biomes:** DXS: deserts & xeric shrublands; MGS: montane grasslands and shrublands; TSGSS: tropical & subtropical grasslands, savannas & shrublands; TSDBF: tropical & subtropical dry broadleaf forests; TSMBF: tropical & subtropical moist braodleaf forests.

**Soils:** LP: leptosols; RG: regosols; CM: cambisols; AR: arenosols; AN: andosols; FR: ferralsols; PH: phaeozems; AC: acrisols; LV: luvisols; FL: fluvisols;

The most frequent soil types found in the study area were those with limited rooting due to shallowness or stoniness, as the leptosols (present in 15/18 productive areas), and relatively young soils, as the regosols (9/18), or with no significant profile development, as the cambisols (9/18). These soils are present in most of the productive areas of Peru, Bolivia, and Ecuador. Andosols soils, derived from volcanic ashes, are also common in Ecuador productive areas. Additionally, soils considered as transitional to a more humid climate, like phaeozems, with more accumulation of organic matter, are also frequently found in the flatter slopes of productive areas.

Demographic, socioeconomic and environmental conditions are distributed differently in each country, with the greatest contrast between the Andean countries and Brazil, followed by the Peruvian coastal productive areas:

- *Brazil* (2 productive areas): Pedra Branca has less than 50 thousand inhabitants, with almost half of the population living in rural areas, and higher rates of poverty. Feira de Santana has a population of more than 500 thousand inhabitants, with most of them living in urban areas, and average poverty rates. Both areas share one type of biome (deserts & xeric shrublands) and have similar climate factors, as well as low altitudes and slopes.
- *Bolivia* (2 productive areas): Franz Tamayo has a population of fewer than 20 thousand inhabitants, is mostly a rural area, and has high poverty rates compared to the country average. Andres Ibanez presents the opposite profile, with close to 2 million inhabitants, a mostly urban population, and low poverty rates. Both areas present one type of biome (tropical & subtropical dry broadleaf forests) and two types of soil (leptosols and cambisols), but they do not share other similar climate factors.
- *Peru* (11 productive areas): Two different patterns were observed: The first includes the six productive areas of Cajamarca, plus the neighboring area of Otuzco, all with less than 200,000 inhabitants, a high percentage of the population living in rural areas, high rates of poverty, and dry ecosystems in their steep piedmont areas. The second pattern is observed on the coast with three areas (Ascope, Trujillo and Pacasmayo), showing lower rates of rural population and poverty, being closer to sea level and having very dry desertic biomes (less than 100 mm of precipitation a year).
- *Ecuador* (3 productive areas): The population ranges from 45 to 225 thousand inhabitants. Guamote, the least populated area, presents a mostly rural population with high poverty levels, as measured by unsatisfied basic needs. The three areas present the same two types of biomes (montane grasslands & shrublands; tropical & subtropical moist broadleaf forests), similar climate factors and soil types.

#### Risk mapping of plague in Latin America

**Figure 4:**
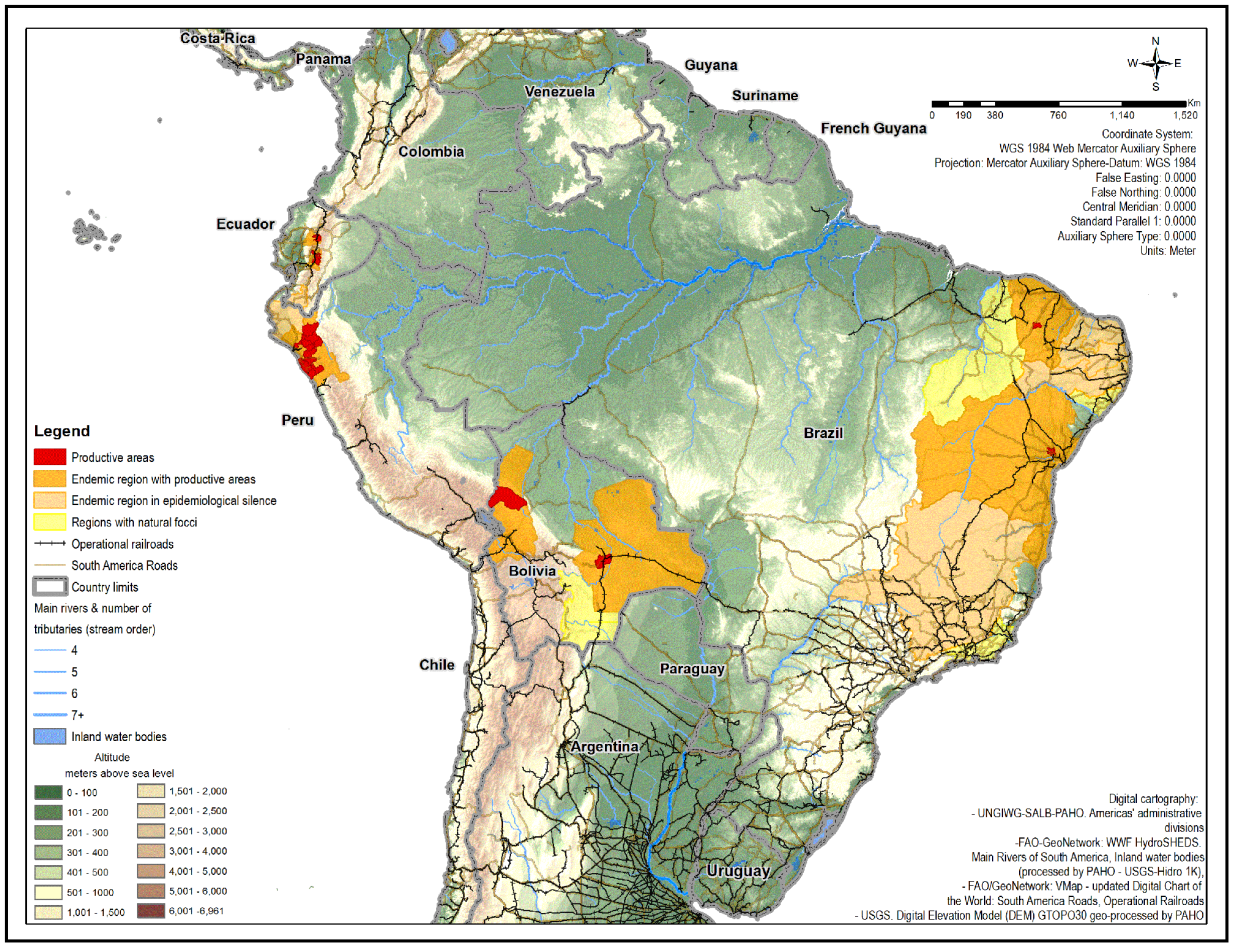
Risk mapping of plague, Latin America.

The mapping of plague risk in Latin America was developed using the epidemiological characterization described in the methodology. The counties where plague still persists were mapped. As well as the regions where plague could potentially emerge or re-emerge, which include: nine endemic regions with productive areas; six endemic regions in epidemiological silence; and five regions with foci without human cases (Supporting Information S5). Plague risk could be considered higher in the 18 areas where the disease still persists, followed by the 70 neighboring counties that surround them. Railroads, land roads, and rivers were added to the map to illustrate the potential interaction between populated areas with and without evidence of plague (Figure 4).

## Discussion

Even though human plague cases are very localized and appear in only 18 of about 13,300 counties of Latin America, the risk is present and unexpected outbreaks could occur. The risk mapping suggested that there are large areas at risk of plague in the endemic countries, not only in productive areas where plague still persists with human cases, but also in neighboring counties where the disease could also emerge or re-emerge from epidemiological silence. Plague natural foci also represent a risk, even if these areas are currently not producing human cases. Attention should also be drawn to regions where plague cases are considered to be in the window of re-emergence or where there is evidence that the disease persists in nature through transmission among susceptible rodents and flea fauna and where potential transmission to humans could occur.

Plague foci have been described in Argentina and Venezuela in previous decades, but no recent information has been found about active foci and no human cases have been reported since the 1950s and 60s. However, according to the literature review, these countries are close to the window of possible re-emergence [1], [2].

The four countries considered as endemic for plague (Bolivia, Brazil, Ecuador, Peru) are implementing surveillance and control actions in animals; however, for most of them, plague is no longer a disease with visibility in the current epidemiological context. Even though only a few cases were reported in the last decade, the risk is still present and an unexpected outbreak could occur, as it had happened in La Libertad, Peru in 2010, where two nosocomial infections of pneumonic plague related to the index case occurred [67]. Another example of an outbreak with pneumonic cases presenting high mortality took place in a remote Andean community in Ecuador in 1998 [68]. A further disaggregated risk evaluation is recommended, including identification of active foci and possible interactions among areas where plague could emerge or re-emerge.

Demographic and poverty patterns were observed in 11 of 18 productive areas, presenting less than 200,000 inhabitants, more than 40% of rural population and high poverty rates. This is in accordance with the existing literature that shows that in the Region, plague affects poorer populations in rural settings [2], [14], [31]. In 2009, plague was included in the list of selected neglected diseases and other poverty-related infections targeted for elimination by PAHO with member countries' agreement [69]. The goal is zero mortality and no domiciliary outbreaks [69]. Cases of human plague affect mostly vulnerable populations, supporting the regional commitment of elimination of neglected diseases and other poverty-related infections. In the Americas, plague is not only considered a poverty-related neglected infection, but also an epidemic-prone disease of potential international concern within the IHR.

Behavioral factors and cultural practices might also impact the risk of human plague. In the Andean subregion, the most affected one, the cultural habits of raising guinea pigs inside homes and preparing them for cooking are risk factors for plague outbreaks [4]. In addition, household hygiene and living conditions are other key factors that influence plague prevalence among humans. Poor sanitation, mostly the accumulation of garbage, as well as the storage of harvested crops inside homes, favor rodent reproduction by providing them with a source of food and at the same time facilitate transmission of the disease to humans [4], [40], [42]. Furthermore, human-to-human transmission, especially among families, could also be sustained by the custom of many Andean countries of holding funeral wakes and offering the deceased's clothing to relatives [24], [37]. Plague risk could also differ by region, depending on the reservoir and vector present locally. For example, *Cavia porcellus* (the type of guinea pig used for cooking) is an important reservoir for the Andean Area, but it is not present in Brazil.

One of the limitations of this study is that we used existing information without conducting a field study. For this reason, it was not possible to determine the exact geographic location of the cases, whether the cases were imported or transmitted locally through animals and fleas, or the locations of active foci. Regarding the cases in Trujillo, Peru, interventions and epidemiological investigations have been conducted and recorded in publications [67], [70]. The outbreak started in Ascope, La Libertad in 2009, after 12 years of epidemiological silence in the area and subsequently, secondary cases occurred in Trujillo [67]. In the Americas, there are no secondary data available for most of the reported cases of plague, in order to determine whether the cases were imported or transmitted locally. A few productive areas have large populations and are mostly urban, for this reason, more disaggregated information is required to determine the exact location of the cases. However, this also suggests that plague remains close to large urban areas.

Previous research has explored the link between plague occurrence and environmental variables [23], [25], [26], [28], [29], [71], [72]. Geographic distribution patterns and their associated factors are unclear [73]. Nonetheless, several studies explored altitude and topography as predictors of transmission [24], [25]. Seasonal variation has also been used to understand the dynamics of vectors and hosts in relation to alternating cycles of temperature and precipitation [23], [25], [26], [28]. More complex ecological modeling has been done using niche characterization [25], [29]. Still, many of these studies were developed either for specific countries or in other regions and did not comprise a continental scale. They also determined that plague occurs primarily in arid-semiarid or low humidity forest types and fails to persist in moist low land areas [23], [25], [26], [28], [71].

From the selected environmental variables, we could suggest a possible pattern for the Andean countries (Bolivia, Ecuador, and Peru) that needs to be further studied at a larger scale (through a closer approach). Most of the productive areas in the Andean countries have steeper slopes even though average altitude varies. The relationship of slope and plague has been studied before with more of an ecological perspective. In China, studies of Himalayans marmot plague foci showed that the most appropriate gradient for marmot burrows is between 5°–15°, and that the number of burrows decreases as the slope increases [74]. Steeper slopes were observed in Andean countries. In our study, 12/18 of the productive areas presented average altitudes higher than 1,300 meters above sea level. Higher altitudes were found in previous studies [24], [25], [71]. However, Brazil shows contrasting patterns; its productive areas are flatter and lower, recording slopes of 9 degrees of inclination and maximum altitudes of 805 meters above sea level.

Elevational variation in the appearance of plague in the Andean countries could be related to the disease persisting long-term in native rodents and fleas at higher elevations, but occasionally spreading to village rat (*Rattus or Rattus norvegicus*) populations at lower elevations. This may occur following widespread plague epizootics in wild rodents at higher elevations or perhaps through transport of infected guinea pigs and their fleas from higher elevations to markets in lower elevations. Movements of human cases with fleas (*Pulex irritans*) in their clothes could also pose some plague risk to lower elevation sites under certain circumstances. An infected person could be a source for pneumonic outbreaks at lower elevations that are far remote from the more stable mountain plague foci.

In a more integrated perspective, altitude, slope, aspect, and compound topographic index were used to characterize an Environmental Niche Model for sub-Saharan Africa, suggesting a more integrated measurement of landscape conditions than the mere altitude [29].

All of the productive areas show the presence of a drier biome within their territory, from desert and xeric shrublands to tropical and subtropical dry broadleaf forests, including biomes with montane grasslands and shrublands incapable to sustain the presence of trees and located in more mountainous zones. Nevertheless, most counties have areas with arid and semiarid conditions. As the productive areas are located in an inter-tropical zone, 11 out of 18 counties have a humid biome, tropical and subtropical moist broadleaf forests, present mostly in their flatter areas.

In Latin America, 15 of the 18 productive areas present shallow/stoniness or relatively young soils with little profile development that are widely spread in arid and semi-arid areas and in mountain regions. Certain research assessing the potential role of soil in plague epidemiology showed that plague bacterium can survive at least 24 days in contaminated ground under natural conditions [73]. Furthermore, *Y. pestis* biotype *Orientalis* may remain viable and fully virulent after 40 weeks in soil [75]. Other studies have revealed that plague reservoirs have significant correlation with subsoil and topsoil characteristics such as texture, mineral contents, and pH. Plague vectors' occurrence presented significant correlation with soil depth, and mineral and organic content [76]. An additional study has demonstrated a geographic relationship between soil distribution and disease outbreaks [77].

Soil analysis may give a broader explanation about *Y. pestis* persistence, transmission during enzootic and epizootic cycles, or bioremediation after a natural or intentional exposure [22]. Control plans may possibly include information on the association between soil properties and plague reservoirs and vectors, in order to have a more complete environmental characterization.

The information that 70 counties were identified as neighbors of productive areas could be used for future risk-based studies, to determine if they present the same environmental conditions that would be favorable for plague emergence. In addition, this information could also be used in National Plans for strengthening prevention measures and control strategies. Most of Peru, Ecuador, and Brazil's productive areas are away from international borders. In Bolivia, however, Franz Tamayo shares a border with the Peruvian region of Puno. In Peru, where plague persists in 11 counties, the analysis of 12 years of evidence of plague cases in humans suggests a geographic cluster of persistence.

Most of the recent cases in Bolivia were reported in the last decade in Apolo, Franz Tamayo, a small rural village at high altitude that is well known as plague foci. The recent cases in Andres Ibanez, a highly populated area in the region of Santa Cruz, were reported as suspected. However, Santa Cruz has presented foci in previous decades. In Bolivia, the counties have extensive areas and the exact location of the suspected cases is unknown. For this reason, a more precise analysis is necessary at the third sub-national level or lower, and additional studies about the epidemiological situation of plague in Bolivia are needed.

The review of the historical presence of plague in the Americas demonstrated the importance of the disease in the region. More than half of the countries in Latin America (14 of 25 of countries) faced at least one plague event with human cases since the end of the 19^th^ century. The historic spread of plague inside countries following trade and transportation is a lesson of great value, particularly in the context of the IHR [3], [10], [24]. Further risk analysis could be carried out to evaluate with more precision the epidemiological situation of possible risk areas close to large cities. The risk mapping shows that potential interaction by railroads, rivers, and roads may be occurring between productive areas and other zones, including highly populated cities. Neighboring counties need to be better evaluated, especially existing trade mechanisms that could potentially introduce rodents with infected fleas into new areas. In the 1990s, a plague outbreak occurred in a populated city in India, creating major public health concern, widespread panic, worldwide apprehension, and severe economic losses for India [78].

Since this was a retrospective study, we suggest that the countries develop more detailed mapping of their productive areas, silent areas, and foci, using the same definitions and methodology to evaluate the risk.

Little is known about the dynamics of plague in its natural reservoir and about changing risk for humans [5]. The environmental variables in this study were selected for an exploratory purpose and to present a brief profile of the productive areas. Zones with the same environmental conditions in productive and neighboring counties may be at higher risk of plague emergence. However, this information was not disaggregated enough to geo-reference cases more precisely in order to perform this analysis. This information could be used as a stepping-stone for future studies with local participation that, within the wider context of a risk-based approach, could include the exact geographic coordinates of cases and the epidemiological investigation that was performed by the national authorities.

Plague continues to be a public health threat with epidemic potential for the Americas, in spite of the low number of human cases and its persistence in fewer than 20 regions. Plague remains a neglected disease that could be addressed within the context of the One Health vision, where animals, humans, and ecosystems interrelate, and multidisciplinary teams and intersectoral collaboration is needed.

The principal conclusion of the International Meeting of Plague Experts in Latin America held by PAHO in Lima, Peru in 2013 was that the preparation of a Regional Strategic Plan on Plague is crucial (PAHO, unpublished data). An “ad hoc” committee was formed during the meeting with representatives from every endemic country in Latin America and PAHO as the secretary. The importance of identifying risk areas and strengthening the first level of care in these areas will be emphasized in the Plan, as well as detection, case management, community surveillance, and intersectoral collaboration for prevention and response of outbreaks. The analysis presented in this study could be used as evidence for decision-making in support of such a Plan to identify higher risk areas, and to prioritize areas for intervention, as well as to identify future studies to be conducted by the countries with international collaboration, when desired.

Areas with different risk characterizations need specific actions of surveillance, prevention, and preparedness to be ready to respond to possible outbreaks. This Plan will aim to strengthen country programs and support the cooperation among countries in their common vision and goal to predict, prevent, detect, and respond to plague events.

## Supporting Information

Supporting Information S1 **Countries with presence of cases of human plague in Latin America, 1899–2012.**
(DOCX)

Supporting Information S2 **Excel database of countries with presence of cases of human plague in Latin America, 1899–2012.**
(XLSX)

Supporting Information S3 **Data source by country.**
(DOCX)

Supporting Information S4 **Table S1. Demographic and socioeconomic variables and sources of information. Table S2. Environmental variables created from original sources.**
(DOCX)

Supporting Information S5 **Endemic regions: (1) with productive areas; (2) in epidemiological silence; (3) with natural foci without human cases, Latin America, 1980–2012.**
(DOCX)

Supporting Information S6 **Alternative language abstract – Spanish.**
(DOCX)
