# Supplementary material for: Where Does Human Plague Still Persist in Latin America?
Source: PLoS Negl Trop Dis. 2014 Feb 6;8(2):e2680. doi: 10.1371/journal.pntd.0002680 (PMC3916238; doi:10.1371/journal.pntd.0002680)
Supplement: Supporting Information S1 — Countries with presence of cases of human plague in Latin America, 1899–2012. (DOCX) [file pntd.0002680.s001.docx]

**Supporting Information S1**

**Table 1. Countries with presence of cases of human plague in Latin America, 1899 – 2012.**

Table 1a. 1899 - 1949

| **Year** | **Countries** | | | | | | | | | | | | |
| --- | --- | --- | --- | --- | --- | --- | --- | --- | --- | --- | --- | --- | --- |
|  | **Argentina** | **Bolivia** | **Brazil** | **Chile** | **Cuba** | **Ecuador** | **Mexico** | **Panama** | **Paraguay** | **Peru** | **Puerto Rico** | **Uruguay** | **Venezuela** |
| **1899** | *X^c^ |  | *X^a,b,c^ |  |  |  |  |  | *X^a^_#_ |  |  |  |  |
| **1900** | X^a^ |  | ○^b^ |  |  |  |  |  |  |  |  |  |  |
| **1901** | ○^c^ |  | ○^b^ |  |  |  |  |  |  |  |  | *X^a^ |  |
| **1902** | ○^c^ |  | ○^b^ |  |  |  | *X^a^ |  |  |  |  |  |  |
| **1903** | ○^c^ |  | ○^b^ | *X^a^ |  |  |  |  |  | *X^a,b^ |  |  |  |
| **1904** | ○^c^ |  | ○^b^ |  |  |  |  |  |  | X^c^ |  |  |  |
| **1905** | ○^c^ |  | ○^b^ |  |  |  |  | *X^a^_#_ |  | X^c^ |  |  |  |
| **1906** | ○^c^ |  | X^b,c^ |  |  |  |  |  |  | X^c^ |  |  |  |
| **1907** | ○^c^ |  | X^b,c^ |  |  |  |  |  |  | X^c^ |  |  |  |
| **1908** | ○^c^ |  | ○^b^ |  |  | *X^a,b^ |  |  |  | X^c^ |  |  | *X^a^ |
| **1909** | ○^c^ |  | ○^b^ |  |  | X^b^ |  |  |  | X^c^ |  |  | ○^c^ |
| **1910** | ○^c^ |  | ○^b^ |  |  | ○^b^ |  |  |  | X^c^ |  |  | X^b^ |
| **1911** | ○^c^ |  | ○^b^ |  |  | ○^b^ |  |  |  | X^c^ |  |  | X^b^ |
| **1912** | ○^c^ |  | ○^b^ |  | *X^a^ | ○^b^ |  |  |  | X^c^ | *X^a^ |  | ○^c^ |
| **1913** | X^c^ |  | ○^b^ |  |  | X^b^ |  |  |  | X^c^ |  |  | ○^c^ |
| **1914** | ○^c^ |  | ○^b^ |  |  | ○^b^ |  |  |  | X^c^ |  |  | X^b^ |
| **1915** | ○^c^ |  | ○^b^ |  | X^a^_#_ | ○^b^ |  |  |  | X^c^ |  |  | ○^c^ |
| **1916** | ○^c^ |  | ○^b^ |  |  | X^b^ |  |  |  | X^c^ |  |  | ○^c^ |
| **1917** | ○^c^ |  | ○^b^ |  |  | ○^b^ |  |  |  | X^c^ |  |  | ○^c^ |
| **1918** | ○^c^ |  | ○^b^ |  |  | ○^b^ |  |  |  | X^c^ |  |  | ○^c^ |
| **1919** | ○^c^ |  | ○^b^ |  |  | ○^b^ |  |  |  | X^c^ |  |  | X^b^ |
| **1920** | ○^c^ |  | ○^b^ |  |  | ○^b^ |  |  |  | X^c^ |  |  | ○^c^ |
| **1921** | ○^c^ | *X^a,b,c^ | ○^b^ |  |  | X^b^ |  |  |  | X^c^ | X^a^_#_ |  | ○^c^ |
| **1922** | ○^c^ | X^b,c^ | ○^b^ |  |  | ○^b^ |  |  |  | X^c^ |  |  | ○^c^ |
| **1923** | ○^c^ |  | ○^b^ |  |  | ○^b^ | X^a^_#_ |  |  | X^c^ |  |  | ○^c^ |
| **1924** | ○^c^ |  | ○^b^ |  |  | ○^b^ |  |  |  | X^c^ |  |  | ○^c^ |
| **1925** | ○^c^ |  | ○^b^ |  |  | ○^b^ |  |  |  | ○^b^ |  |  | ○^c^ |
| **1926** | ○^c^ |  | ○^b^ |  |  | X^b^ |  |  |  | ○^b^ |  |  | ○^c^ |
| **1927** | ○^c^ |  | ○^b^ |  |  | ○^b^ |  |  |  | ○^b^ |  |  | ○^c^ |
| **1928** | ○^c^ | X^a,b,c^ | X^b^ |  |  | ○^b^ |  |  |  | X^c^ |  |  | X^a,b^ |
| **1929** | ○^c^ |  | X^b^ |  |  | X^b^ |  |  |  | X^c^ |  | X^a^ | ○^c^ |
| **1930** | ○^c^ |  | ○^b^ | X^a^ |  | X^a,b^ |  |  |  | ○^b^ |  |  | ○^c^ |
| **1931** | X^a,b,c^ |  | ○^b^ | X^a^_#_ |  | ○^b^ |  |  |  | ○^b^ |  |  | ○^c^ |
| **1932** | X^b,c^ | X^c^ | ○^b^ |  |  | ○^b^ |  |  |  | ○^b^ |  | X^a^_#_ | X^b^ |
| **1933** | X^b,c^ | X^b,c^ | ○^b^ |  |  | X^b^ |  |  |  | ○^b^ |  |  | X^b^ |
| **1934** | X^a,b,c^ | X^b,c^ | X^b,c^ |  |  | ○^b^ |  |  |  | ○^b^ |  |  |  |
| **1935** | X^a,b,c^ | X^b,c^ | X^b,c^ |  |  | X^a,b^ |  |  |  | ○^b^ |  |  |  |
| **1936** | X^a,b,c^ | X^b^ | X^a,b,c^ |  |  | X^b^ |  |  |  | ○^b^ |  |  |  |
| **1937** | X^b,c^ | X^b,c^ | X^b,c^ |  |  | X^b,c^ |  |  |  | ○^b^ |  |  |  |
| **1938** | X^b,c^ | X^b,c^ | X^b,c^ |  |  | X^b,c^ |  |  |  | ○^b^ |  |  |  |
| **1939** | X^b,c^ | X^b,c^ | X^b,c^ |  |  | X^b^ |  |  |  | X^c^ |  |  | X^a,c^ |
| **1940** | X^b,c^ | X^b,c^ | X^b^ |  |  | X^b^ |  |  |  | ○^b^ |  |  |  |
| **1941** | X^b,c^ | X^c^ | X^b^ |  |  | X^b^ |  |  |  | ○^b^ |  |  |  |
| **1942** | X^b,c^ | X^c^ | X^b^ |  |  | X^b^ |  |  |  | ○^b^ |  |  |  |
| **1943** | X^b,c^ | X^b,c^ | X^b^ |  |  | X^b^ |  |  |  | ○^b^ |  |  | X^b,c^ |
| **1944** | X^b,c^ | X^b,c^ | X^b^ |  |  | X^b^ |  |  |  | ○^b^ |  |  |  |
| **1945** | X^c^ | X^b,c^ | X^b^ |  |  | X^b^ |  |  |  | ○^b^ |  |  |  |
| **1946** | X^b,c^ | X^b,c^ | X^b^ |  |  | X^b^ |  |  |  | X^c^ |  |  |  |
| **1947** | X^b,c^ | X^c^ | X^b^ |  |  | X^b^ |  |  |  | X^c^ |  |  |  |
| **.1948** | X^b^ | X^c^ | X^b^ |  |  | X^b^ |  |  |  | X^c^ |  |  | X^b,c^ |
| **1949** | X^c^ | X^c^ | X^b^ |  |  | X^b^ |  |  |  | X^c^ |  |  | X^b,c^ |

**Legend:**

X: Presence of plague documented on that year; ○: Presence of plague documented on the period (consolidated data); *: First case reported; #: Last case reported;

**Sources:**

1. Moll AA, O'Leary SB (1940) Plague in the Americas: an Historical and Quasi-Epidemiological Survey. Boletin de la Oficina Sanitaria Panamericana 19: 576-584.
2. Pollitzer R (1954) Plague. Geneva: World Health Organization.
3. PAHO (1965) Plague in the Americas. Washington, D.C.: Pan American Health Organization.

Table 1b. 1950 – 1979

| **Year** | **Countries** | | | | | | |
| --- | --- | --- | --- | --- | --- | --- | --- |
|  | **Argentina** | **Bolivia** | **Brazil** | **Ecuador** | **El Salvador** | **Peru** | **Venezuela** |
| **1950** |  | X^b^ | X^a^ | X^a,b^ |  | X^b^ | X^a,b^ |
| **1951** | X^a,b^ | X^b^ | X^a^ | X^a^ |  | X^b^ | X^a,b^ |
| **1952** | X^b^ | X^b^ | X^b^ | X^a^ |  | X^b^ |  |
| **1953** |  | X^b^ | X^b^ | X^b^ |  | X^b^ | X^b^ |
| **1954** |  | X^b,c^ | X^c^ | X^b,c^ |  | X^b,c^ |  |
| **1955** |  | X^b,c^ | X^c^ | X^b,c^ | *X^c^_#_ | X^b,c^ |  |
| **1956** |  | X^b,c^ | X^c^ | X^b,c^ |  | X^b,c^ | X^b,c^ |
| **1957** |  | X^b^ | X^c^ | X^b,c^ |  | X^b,c^ |  |
| **1958** | X^b,c^_#_ | X^b^ | X^c^ | X^b,c^ |  | X^b,c^ |  |
| **1959** |  | X^b^ | X^c^ | X^b,c^ |  | X^b,c^ |  |
| **1960** |  | X^b,c^ | X^c^ | X^b,c^ |  | X^b,c^ | X^b^ |
| **1961** |  | X^b,c^ | X^c^ | X^b,c^ |  | X^b,c^ | X^b,c^ |
| **1962** |  | X^b^ | X^c^ | X^b,c^ |  | X^b,c^ | X^b^ |
| **1963** |  | X^b,c^ | X^c^ | X^b,c^ |  | X^b,c^ | X^c^_#_ |
| **1964** |  | X^c^ | X^c^ | X^c^ |  | X^c^ |  |
| **1965** |  | X^c^ | X^c^ | X^c^ |  | X^c^ |  |
| **1966** |  | X^c^ | X^c^ | X^c^ |  | X^c^ |  |
| **1967** |  | X^c^ | X^c^ | X^c^ |  | X^c^ |  |
| **1968** |  | X^c^ | X^c^ | X^c^ |  | X^c^ |  |
| **1969** |  | X^c^ | X^c^ | X^c^ |  | X^c^ |  |
| **1970** |  | X^c,d^ | X^c,d^ | X^c,d^ |  | X^c,d^ |  |
| **1971** |  | X^d^ | X^c,d^ | X^c,d^ |  | X^c,d^ |  |
| **1972** |  |  | X^c,d^ | X^c,d^ |  | X^c,d^ |  |
| **1973** |  |  | X^c,d^ | X^c,d^ |  | X^c,d^ |  |
| **1974** |  | X^c,d^ | X^c,d^ |  |  | X^c,d^ |  |
| **1975** |  | X^c,d^ | X^c,d^ |  |  | X^c,d^ |  |
| **1976** |  | X^c,d^ | X^c,d^ | X^c,d^ |  | X^c,d^ |  |
| **1977** |  | X^c,d^ | X^c,d^ |  |  |  |  |
| **1978** |  | X^c,d^ | X^c^ |  |  | X^c,d^ |  |
| **1979** |  | X^c,d^ |  |  |  |  |  |

**Legend:**

X: Presence of plague documented on that year; *: First case reported; #: Last case reported;

**Sources:**

1. Pollitzer R (1954) Plague. Geneva: World Health Organization.
2. PAHO (1965) Plague in the Americas. Washington, D.C.: Pan American Health Organization.
3. WHO (2000) WHO Report on Global Surveillance of Epidemic-prone Infectious Diseases. Geneva: World Health Organization. 25-37 p.
4. PAHO (1981) Status of Plague in the Americas, 1970-1980. Epidemiological Bulletin 2: 5-8.

Table 1c. 1980 – 2012

| **Year** | **Countries** | | | |
| --- | --- | --- | --- | --- |
|  | **Bolivia** | **Brazil** | **Ecuador** | **Peru** |
| **1980** | X^a,b^ | X^a,b^ |  |  |
| **1981** | X^a,b^ | X^a,b^ | X^a,b^ | X^a,b^ |
| **1982** | X^a,b^ | X^a,b^ | X^b^ | X^a,b^ |
| **1983** | X^a,b^ | X^a,b^ | X^a,b^ | X^a,b^ |
| **1984** | X^a,b^ | X^a,b^ | X^a,b^ | X^a,b^ |
| **1985** |  | X^a,c^ | X^a,c^ | X^a,c^ |
| **1986** | X^a,c^ | X^a,c^ |  |  |
| **1987** | X^a,c^ | X^a,c^ |  | X^a,c^ |
| **1988** | X^a,c^ | X^a,c^ |  | X^a,c^ |
| **1989** | X^d^ | X^a,e^ |  |  |
| **1990** | X^a,f^ | X^a,f^ |  | X^a,f^ |
| **1991** |  | X^a,f^ |  | X^f^ |
| **1992** |  | X^a,f^ |  | X^a,f^ |
| **1993** |  | X^g^ |  | X^a,f^ |
| **1994** |  | X^a,g^ |  | X^a,h^ |
| **1995** |  | X^a,g^ |  | X^a,h^ |
| **1996** | X^a,h^ | X^a,g^ |  | X^a,h^ |
| **1997** | X^a,h^ | X^g,i^ | X^i^ | X^a,h^ |
| **1998** | X^i^ | X^g,i^ | X^a,j^ | X^a,i^ |
| **1999** |  | X^g,i^ | X^j^ | X^i,k^ |
| **2000** | X^l^ | X^g,i^ | X^m^ | X^i,k^ |
| **2001** |  |  | X^m^ | X^k,n^ |
| **2002** |  |  |  | X^k,n^ |
| **2003** |  |  |  | X^k^ |
| **2004** |  |  | X^m,n^ | X^k,n^ |
| **2005** |  | X^n,o^ | X^m^ | X^k,n^ |
| **2006** |  |  |  | X^k,n^ |
| **2007** |  |  |  | X^k,n^ |
| **2008** | X^l !^ |  | X^m,n^ | X^k,n^ |
| **2009** |  |  |  | X^k,p^ |
| **2010** | X^l !^ |  |  | X^k,p^ |
| **2011** |  |  |  | X^k,p^ |
| **2012** | X^l !^ |  |  | X^k,p^ |

**Legend:**

X: Presence of plague documented on that year; !: Suspected case;

**Sources:**

1. WHO (2000) WHO Report on Global Surveillance of Epidemic-prone Infectious Diseases. Geneva: World Health Organization. 25-37 p.
2. PAHO (1986) Health Conditions in the Americas 1981-1984. Washington D.C.: Pan American Health Organization. 1: 427 p.
3. PAHO (1990) Health Conditions in the Americas. Washington D.C.: Pan American Health Organization. 1: 519 p.
4. PAHO (1994) Health Conditions in the Americas. Washington D.C.: Pan American Health Organization. 2: 478 p.
5. PAHO (1989) Diseases Subject to the International Health Regulations. Epidemiological Bulletin 10: 16.
6. PAHO (1994) Health Conditions in the Americas. Washington D.C.: Pan American Health Organization. 1: 447 p.
7. Brazil, Ministry of Health (2008) Manual de Vigilância e Controle da Peste. Brasília: Secretaria de Vigilância em Saúde: 95 p.
8. PAHO (1998) Health in the Americas. Washington D.C.: Pan American Health Organization. 1: 359 p.
9. PAHO (2002) Health in the Americas. Washington D.C.: Pan American Health Organization. 1: 449 p.
10. PAHO (2002) Health in the Americas. Washington D.C.: Pan American Health Organization. 2: 593 p.
11. Peru, Ministry of Health (2013) Casos de peste confirmados por años 1995-2013 - Sala Situacional No.18. Dirección Regional de Epidemiologia.
12. Bolivia, Ministry of Health and Sports (2012) Sistema Nacional de Información en Salud y Vigilancia Epidemiológica. Available online: http://www.sns.gob.bo/snis/default.aspx. Accessed on 7 December 2012.
13. Pezantes C. Presentation of the epidemiological situation of plague in Peru. In International Meeting of Plague Experts in Latin America; Lima, Peru: January 2013. Ministry of Health of Ecuador (unpublished data).
14. PAHO (2010) Health Information and Analysis Project. Regional Core Health Data Initiative. Available online: http://www1.paho.org/English/SHA/coredata/tabulator/newTabulator.htm. Accessed on 13 November 2012.
15. Brazil, Ministry of Health. Departamento de Informática do Sistema Único de Saúde (DATASUS). Available online: http://dtr2004.saude.gov.br/sinanweb/tabnet/dh?sinan/peste/bases/pestebr.def. Accessed on 14 November 2012.
16. Peru, Ministry of Health (2013) Situación de Peste en La Libertad. Boletín Epidemiológico 22: 143-145.
