# Supplementary material for: Where Does Human Plague Still Persist in Latin America?
Source: PLoS Negl Trop Dis. 2014 Feb 6;8(2):e2680. doi: 10.1371/journal.pntd.0002680 (PMC3916238; doi:10.1371/journal.pntd.0002680)
Supplement: Supporting Information S3 — Data source by country. (DOCX) [file pntd.0002680.s003.docx]

**Supporting Information S3**

**Data source by country**

*Bolivia:* Only the Ministry of Health online database was used [1]. Cases in 2008 and 2010 were reported as suspected. Information was available for all years in the studied series.

*Brazil:* For this country four different sources were used: the Ministry of Health online database [2], two official publications [3,4] and unpublished information obtained through country officials confirming the name of the Bahia’s county case in 2000 (Brazil, Ministry of Health, Secretary of Health Surveillance, unpublished data). There was available information for all years in the studied series.

*Ecuador:* For Ecuador no Epidemiological Bulletin was found available online, for this reason the sources used to identify the counties with presence of plague cases were previous information sent by country officials to PAHO (Ecuador, Ministry of Heath, unpublished data) and the presentation from the country representative during the “International Meeting of Plague Experts in Latin America” (Pezantes C, unpublished). Two cases in Ecuador in 2001 were not possible to define the counties.

*Peru:* In order to identify the counties where cases were present several sources were used [5-10] (Peru, Ministry of Health, unpublished data). The main source searched was the Epidemiological Bulletins from the Ministry of Health, however Bulletins were available online starting from 2004 and not all of them reported the name of counties, for this data. For 2000, 2001, 2003 and 2006, since no Epidemiological Bulletins and official documents were available with the names of the counties; the information was obtained through unpublished information sent by country officials to PAHO in 2002, 2005 and 2007 reporting cases of plague (PAHO, unpublished data). For the total number of cases per year a more recent publication with consolidate information was used for the period [11].

*Source for Figure 3:* The following sources were also used to gather information about the consolidated number of human cases of plague by country in Latin America from 2000 to 2012 (Figure 3 in the main text): [1,11] (Pezantes C, unpublished), (Ecuador, Ministry of Heath, unpublished data).

**References**

1. Bolivia, Ministry of Health and Sports (2012) Sistema Nacional de Información en Salud y Vigilancia Epidemiológica. Available online: http://www.sns.gob.bo/snis/default.aspx. Accessed on 7 December 2012.
2. Brazil, Ministry of Health Departamento de Informática do Sistema Único de Saúde (DATASUS). Available online: http://dtr2004.saude.gov.br/sinanweb/tabnet/dh?sinan/peste/bases/pestebr.def. Accessed on 14 November 2012.
3. Brazil, Ministry of Health (2010) Perfil Epidemiológico da Peste no Brasil 2000-2009. Brasília: Secretaria de Vigilância em Saúde.
4. Brazil, Ministry of Health (2008) Manual de Vigilância e Controle da Peste. Brasília: Secretaria de Vigilância em Saúde. 95 p.
5. Peru, Ministry of Health (2003) Sistema Nacional de Vigilancia Epidemiológica - Anuario 2002. Lima, Peru: Dirección General de Epidemiología y Red Nacional de Epidemiología. 140-143 p.
6. Peru, Ministry of Health (2004) Enfermedades de Notificación Obligatoria Según Reglamento Sanitario Internacional. Boletín Epidemiológico 13: 1-8.
7. Peru, Ministry of Health (2005) Tendencia de las enfermedades y daños. Boletín Epidemiológico 14: 1-16.
8. Peru, Ministry of Health (2007) Enfermedades Zoonóticas - Peste. Boletín Epidemiológico 16: 697-750.
9. Peru, Ministry of Health (2008) Enfermedades Zoonóticas - Peste. Boletín Epidemiológico 17: 962-1041.
10. Peru, Ministry of Health (2013) Situación de Peste en La Libertad. Boletín Epidemiológico 22: 143-145.
11. Peru, Ministry of Health (2013) Casos de peste confirmados por años 1995-2013 - Sala Situacional No.18. Dirección Regional de Epidemiologia.
