# Supplementary material for: Where Does Human Plague Still Persist in Latin America?
Source: PLoS Negl Trop Dis. 2014 Feb 6;8(2):e2680. doi: 10.1371/journal.pntd.0002680 (PMC3916238; doi:10.1371/journal.pntd.0002680)
Supplement: Supporting Information S4 — Table S1. Demographic and socioeconomic variables and sources of information. Table S2. Environmental variables created from original sources. (DOCX) [file pntd.0002680.s004.docx]

**Supporting Information S4**

**Table 1. Demographic and socioeconomic variables and sources of information.**

| **Demographic and socioeconomic variables** | **Sources** |
| --- | --- |
| Bolivia, total population (2010)^*^ | Bolivia, Instituto Nacional Estadística [1] |
| Bolivia, rural population (2001) | Bolivia, Instituto Nacional Estadística [2] |
| Bolivia, extreme poverty (2001) | Bolivia, Instituto Nacional Estadística [3,4] |
| Brazil, total population (2010) | Brazil, Instituto Brasileiro de Geografia e Estatística [5] |
| Brazil, rural population (2010) | Brazil, Instituto Brasileiro de Geografia e Estatística [5] |
| Brazil, poverty headcount ratio at national poverty line (country) (2004) | World Bank [6] |
| Brazil, poverty incidence (2003) | Brazil, Instituto Brasileiro de Geografia e Estatística [7] |
| Ecuador, total population (2010) | Ecuador, Instituto Nacional de Estadística e Censos [8] |
| Ecuador, rural population (2010) | Ecuador, Instituto Nacional de Estadística e Censos [8] |
| Ecuador, poverty based on unsatisfied basic needs (2010) | Ecuador, Instituto Nacional de Estadística e Censos [9] |
| Peru, total population (2007) | Peru, Instituto Nacional de Estadística e Informática [10] |
| Peru, rural population (2007) | Peru, Instituto Nacional de Estadística e Informática [10] |
| Peru, extreme poverty (2009) | Peru, Ministry of Health [11]; Peru, Instituto Nacional de Estadística e Informática [12] |

*: Projected population;

**Table 2. Environmental variables created from original sources.**

| **Environmental variables** | **Sources** |
| --- | --- |
| Altitude (meters above sea level) | U.S. Geological Surveys EROS Data Center [13] |
| Slope (degrees 0-90) | U.S. Geological Surveys EROS Data Center [13] |
| Temperature (annual mean temperature^*^) | Hijmans, et al. [14]; WorldClim [15] |
| Precipitation (annual precipitation^#^) | Hijimans, et al [14]; WorldClim [15] |
| Biomes | World Wild Fund [16] |
| Soils groups and types | Food and Agriculture Organization [17]; UNGIWG-SALB-PAHO [18] |

^*^: The mean of all the weekly mean temperatures. Each weekly mean temperature is the mean of that week's maximum and minimum temperature in celsius degrees;

^#^: The sum of all the monthly precipitation estimates in millimeters.

**References**

1. Bolivia, Instituto Nacional Estadística (2011) Poblacion total, proyectada, por sexo, segun provincia y seccion de provincia, 2009-2011. Available online: http://www.ine.gob.bo/indice.aspx?d1=0310&d2=6. Accessed on 06 June 2013.
2. Bolivia, Instituto Nacional Estadística (2001) Poblacion por sexo y area segun departamento, provincia y municipio, censo 2001. Available online: http://www.ine.gob.bo.indice/visualizador.aspx?ah=pC20102.HTM. Accessed on 06 June 2013.
3. Bolivia, Instituto Nacional Estadística (2005) Estadisticas e indicadores sociodemograficos, productivos y financieros por municipio-Departamento de La Paz. Available online: http://www.ine.gob.bo/html/dfid/index.htm. Accessed on 06 June 2013.
4. Bolivia, Instituto Nacional Estadística (2005) Estadisticas e indicadores sociodemograficos, productivos y financieros por municipio-Departamento de Santa Cruz. Available online: http://www.ine.gob.bo/html/dfid/index.htm. Accessed on 06 June 2013.
5. Brazil IBdGeEI (2010) Synopsis of the 2010 Population Census. Available online: http://www.ibge.gov.br/cidadesat/topwindow.htm?1. Accessed on 06 June 2013.
6. WorldBank The World Bank DataBank-Poverty and Inequality Databank. Available online: http://data.worldbank.org/indicator/SI.POV.NAHC/countries/BR?page=1&display=default. Accessed on 11 June 2013.
7. Brazil, Instituto Brasileiro de Geografia e Estatistica (IBGE) (2003) Map of Poverty and Inequality-Brazilian Municipalities. Available online: http://www.ibge.gov.br. Accessed on 06 June 2013.
8. Ecuador, Instituto Nacional de Estadistica e Censos (INEC) (2010) Censo de poblacion y vivienda 2010: Poblacion por area, segun provincia, canton y parroquia de empadronamiento. Available online: http://www.inec/gob.ec/estadisticas/?option=com_content&view=article&id=109&ltemid=88. Accessed on 06 June 2013.
9. Ecuador, Instituto Nacional de Estadistica e Censos (INEC) (2010) Censo de poblacion y vivienda 2010: Poblacion-Necesidades Basicas Insatisfechas NBI Total Nacional. Available online: http://www.inec.gob.ec/estadisticas/?option=com_content&view=article&id=109&ltemid=88. Accessed on 06 June 2013.
10. Peru Instituto Nacional de Estadistica e Informatica (INEI) (2007) Censo Nacionale 2007-Tipo de area. Available online: http://iinei.inei.gob.pe/iinei/RedatamCpv2007.asp?id=ResultadosCensales?ori=C. Accessed on 06 June 2013.
11. Peru, Ministry of Health (2009) Indicadores Basicos de Salud Peru-Indicadores de Determinantes Sociales. Available online: http://www.dge.gob.pe/Asis/indbas/2009/socioeconomico.pdf. Accessed on 11 June 2013.
12. Peru, Direccion Tecnica de Demografia e Indicadores Sociales del Instituto Nacional de Estadistica e Informatica (INEI) (2009) Mapa de Probreza Provincial y Distrital 2009-El Enfoque de la pobreza monetaria. Lima, Peru: Instituto Nacional de Estadistica e Informatica. 288 p.
13. U.S. Geological Survey's (USGS). EROS Center. Elevation Prroducts. Available online: http://eros.usgs.gov/elevation-products Accessed on 25 January 2013.
14. Hijmans RJ, Cameron SE, Parra JL, Jones PG, Jarvis A (2005) Very high resolution interpolated climate surfaces for global land areas. International Journal of Climatology 25: 1965-1978.
15. World Clim - Global Climate Data. Available online: http://www.worldclim.org/. Accessed on 17 May 2013.
16. World Wildlife Fund. Terrestrial Eco-regions (Biomes). Available online: http://worldwildlife.org/biomes. Accessed on 19 April 2013.
17. FAO-Geonetwork (2007) Digital Soil Map of the World. Available online: http://www.fao.org/geonetwork/srv/en/main.home. Accessed on 25 January 2013.
18. UNGIWG-SALB-PAHO (2013) The Second Administrative Level Boundaries (SALB) data set project UN Geographic Information Working Group (UNGIWG). Available online: http://www.unsalb.org/. Accessed on June 2013 (originally co-produced during January - November 2007; processed by PAHO/HSD/IR, - July 2008-March 2013).
