# Supplementary material for: Where Does Human Plague Still Persist in Latin America?
Source: PLoS Negl Trop Dis. 2014 Feb 6;8(2):e2680. doi: 10.1371/journal.pntd.0002680 (PMC3916238; doi:10.1371/journal.pntd.0002680)
Supplement: Supporting Information S5 — Endemic regions: (1) with productive areas; (2) in epidemiological silence; (3) with natural foci without human cases, Latin America, 1980–2012. (DOCX) [file pntd.0002680.s005.docx]

**Supporting Information S5**

**Endemic regions: (1) with productive areas; (2) in epidemiological silence; (3) with natural foci without human cases, Latin America, 1980 – 2012.**

| **Country** | **Regions** | **Endemic regions** | | |
| --- | --- | --- | --- | --- |
|  |  | With productive areas | In epidemiological silence | With natural foci without human cases |
| **Bolivia** |  |  |  |  |
|  | Chuquisaca |  |  | X ^j^ |
|  | La Paz | X ^a^ |  |  |
|  | Santa Cruz | X ^a^ |  |  |
|  | Tarija |  |  | X ^j^ |
| **Brazil** |  |  |  |  |
|  | Alagoas |  |  | X ^h^ |
|  | Bahía | X ^b^ |  |  |
|  | Ceará | X ^b^ |  |  |
|  | Minas Gerais |  | X ^h^ |  |
|  | Paraiba |  | X ^h^ |  |
|  | Peranambuco |  | X ^h^ |  |
|  | Piaui |  |  | X ^h^ |
|  | Rio de Janeiro |  |  | X ^h^ |
|  | Rio Grande do Norte |  | X ^h^ |  |
| **Ecuador** |  |  |  |  |
|  | Chimborazo | X ^c,d^ |  |  |
|  | Cotopaxi | X ^d^ |  |  |
|  | Loja |  | X ^i^ |  |
| **Peru** |  |  |  |  |
|  | Cajamarca | X ^e,f^ |  |  |
|  | La Libertad | X ^f^ |  |  |
|  | Lambayeque | X ^g^ |  |  |
|  | Piura |  | X ^i^ |  |

**Definitions:**

- Productive areas where plague still persists: political/administrative divisions (in this case at the second sub-national level) where evidence of presence of cases of human plague was detected in official documents between 2000 and 2012.
- Endemic regions with productive areas: political/administrative divisions (in this case at the first sub-national level) where evidence of presence of cases of human plague was detected between 2000 and 2012.
- Endemic regions in epidemiological silence: political/administrative divisions (in this case at the first sub-national level) that have reported one or more cases of human plague between 1980 and 1999 and where there is no evidence of presence of cases of human plague between 2000 and 2012.
- Regions with foci: political/administrative divisions (in this case at the first sub-national level) that have been cited in the literature between 1980 and 2012 with one or more foci present and no evidence of human cases in the same period.

**Sources:**

1. Bolivia, Ministry of Health and Sports (2012) Sistema Nacional de Información en Salud y Vigilancia Epidemiológica. Available online: http://www.sns.gob.bo/snis/default.aspx. Accessed on 7 December 2012.
2. Brazil, Ministry of Health (2010) Perfil Epidemiológico da Peste no Brasil 2000-2009. Brasília: Secretaria de Vigilância em Saúde.
3. Pezantes C. Presentation of the epidemiological situation of plague in Peru. In International Meeting of Plague Experts in Latin America; Lima, Peru: January 2013. Ministry of Health of Ecuador (unpublished data).
4. Ecuador, Ministry of Health (2005) Resolver la agenda inconclusa de salud en el Ecuador: Intervención multifactorial para disminuir los factores de riesgo de transmisión de la peste a los pobladores de los focos de riesgo en el Ecuador (unpublished data).
5. Peru, Ministry of Health, PAHO (2008) Plan Integral para la Eliminación de la Peste Humana en Área Endémicas de la Macro Región Norte del Perú 2008-2012. Lima: Ministerio de Salud de Peru & PAHO (unpublished data).
6. Peru, Ministry of Health (2012) Plan Integral de Prevención y Control de Peste La Libertad 2012-2013. Trujillo: Gerencia Regional de Salud La Libertad (unpublished data).
7. PAHO (2002) Number of cases of human plague in 2000. Information sent to the Pan American Health Organization by the Ministry of Health of Peru (unpublished data).
8. Brazil, Ministry of Health (2008) Manual de Vigilância e Controle da Peste. Brasília: Secretaria de Vigilância em Saúde: 95 p.
9. PAHO (1990) Health Conditions in the Americas. Washington D.C.: Pan American Health Organization. 1: 519 p.
10. PAHO (2002) Health in the Americas. Washington D.C.: Pan American Health Organization. 2: 593 p.
