# Supplementary material for: Where Does Human Plague Still Persist in Latin America?
Source: PLoS Negl Trop Dis. 2014 Feb 6;8(2):e2680. doi: 10.1371/journal.pntd.0002680 (PMC3916238; doi:10.1371/journal.pntd.0002680)
Supplement: Supporting Information S6 — Alternative language abstract – Spanish. (DOCX) [file pntd.0002680.s006.docx]

**Supporting Information S6: Alternative language abstract – Spanish**

**Donde Persiste Todavía la Peste Humana en América Latina?**

Maria Cristina Schneider ^1^*; Patricia Najera ^1^; Sylvain Aldighieri ^1^; Deise I. Galan ^1^; Eric Bertherat ^2^; Alfonso Ruiz ^3^; Elsy Dumit ^1^; Jean Marc Gabastou ^1^; Marcos A. Espinal ^1^

^1^  Department of Communicable Diseases and Health Analysis, Pan American Health Organization, 525 23rd. Street NW, Washington, DC 20037, USA; Emails: [schneidc@paho.org](mailto:schneidc@paho.org); [najerapa@paho.org](mailto:najerapa@paho.org); [aldighsy@paho.org](mailto:aldighsy@paho.org); [galand@paho.org](mailto:galand@paho.org); [elsydumit@gmail.com](mailto:elsydumit@gmail.com) ; [gabastouj@paho.org](mailto:gabastouj@paho.org); [espinalm@paho.org](mailto:espinalm@paho.org);

^2^  Unit of Control of Epidemic Diseases, World Health Organization, Avenue Appia 20, 1211 Geneva 27, Switzerland; Email: [bertherate@who.int](mailto:bertherate@who.int);

3 Department of Global Health, University of South Florida, 13201 Bruce B. Downs Blvd. MDC 56, Tampa, FL 33612, USA; Email: [ruizalfo@hotmail.com](mailto:ruizalfo@hotmail.com)

* Corresponding author. Email: [schneidc@paho.org](mailto:schneidc@paho.org);

**Abstracto**

*Antecedentes:* La peste es una enfermedad de potencial epidémico con posible impacto en la salud pública, el comercio internacional y el turismo. Puede emerger y reemerger después de décadas de silencio epidemiológico. Al momento en América Latina, los casos humanos y focos de la peste están presentes en Bolivia, Brasil, Ecuador y Perú.

*Objetivos:* El objetivo de este estudio es identificar donde persisten aún casos de peste humana y colocar en el mapa áreas que pueden estar en riesgo de emerger o reemerger en América Latina. Este análisis proporcionará información basada en evidencia para que los países prioricen las áreas de intervención.

*Métodos*: La evidencia sobre la presencia de la peste se demostró utilizando la información oficial existente de la OMS, la OPS y los Ministerios de Salud. Una base de datos geo-referenciada fue creada para establecer la presencia histórica de la peste por país entre el primer caso registrado en 1899 y el 2012. Las áreas donde la peste persiste fueron analizadas por segundo nivel de las divisiones político/administrativas (condados). Se describieron variables seleccionadas demográficas, socioeconómicas y medioambientales.

*Resultados:* Se encontró que la peste está presente por uno o más años en 14 de 25 países de América Latina (1899-2012). Los focos han persistido en seis países, en dos de los cuales no se tiene informe de casos actuales. Hay evidencia de que los casos humanos de peste aún persisten en 18 condados. Se observaron patrones demográficos y de pobreza en 11 de los 18 condados. Cuatro tipos de biomas son los más comúnmente encontrados, 12/18 tienen una altura superior promedio de 1300 metros sobre el nivel del mar.

*Discusión:* A pesar de que los casos de peste humana están muy localizados, el riesgo está presente y pueden ocurrir brotes inesperados. Los países tienen que hacer el último esfuerzo para eliminar la peste como un problema de salud pública en las Américas. Se recomienda una evaluación de riesgo más desagregada, incluyendo la identificación de los focos y las posibles interacciones entre las áreas donde la peste podría emerger o remerger. Se sugiere una caracterización del ambiente a mayor aproximación.
